# Profil clinique de la dépression post- accident vasculaire cérébral étude transversale descriptive menée au centre de réhabilitation des personnes handicapées de Kinshasa (RD Congo)

**DOI:** 10.11604/pamj.2014.17.109.3296

**Published:** 2014-02-13

**Authors:** Magloire Nkosi Mpembi, Samuel Mampunza Ma Miezi, Thierry Matonda Ma Nzuzi, Victoria Kubuta Massamba, Sévérine Henrard, Marie-Pierre De Partz, André Peeters, Jean Macq, Vincent Dubois, Eric Constant

**Affiliations:** 1 Institut de Recherche Santé et Société, Université Catholique de Louvain, Bruxelles, Belgique; 2 Centre Neuro Psycho Pathologique, Université de Kinshasa, Kinshasa, RD Congo; 3 Research Center in Epidemiology, Biostatistics and Clinical Trials, School of Public Health, Université Libre de Bruxelles (ULB), Bruxelles, Belgique; 4 Unité de neuropsychologie, Cliniques universitaires Saint Luc, Université Catholique de Louvain, Bruxelles, Belgique; 5 Stroke Unit, Cliniques Universitaires Saint Luc, Université Catholique de Louvain, Bruxelles, Belgique; 6 Département de psychiatrie, Cliniques universitaires Saint Luc, Université Catholique de Louvain, Bruxelles, Belgique

**Keywords:** Accident vasculaire cérébral, dépression, Kinshasa, Stroke, depression, Kinshasa

## Abstract

**Introduction:**

L'objectif général poursuivi dans cette étude est de décrire le tableau clinique de la dépression post-accident vasculaire cérébral (DPAVC) à Kinshasa.

**Méthodes:**

Il s'agit d'une étude transversale descriptive portant sur 56 patients suivis pour hémiplégie post-accident vasculaire cérébral au Centre de réhabilitation pour personnes handicapées de Kinshasa (CRPHK) du 1er au 31 août 2011.

**Résultats:**

Au Patient Health Questionnaire (PHQ9), 21. 40 % des patients présentaient une dépression modérée à sévère. A l’échelle de Rankin, 63. 8% des patients étaient capables de marcher sans aide. L'apathie modérée à sévère était présente chez 44. 64%; à l’échelle de sévérité de la fatigue, le score de 12. 7 % des sujets était compatible avec un état dépressif. La dépression était associée à la sévérité des troubles neurologiques, à l'incapacité évaluée avec l’échelle de Rankin, à l'apathie et à une appréciation mauvaise de son propre état de santé par le patient.

**Conclusion:**

La DPAVC est fréquente à Kinshasa parmi les patients en réhabilitation. La fréquence observée est comparable à celles retrouvées dans des travaux antérieurs dans le monde et aux rares travaux publiés en Afrique. La DPAVC est associée de manière significative à la sévérité des troubles neurologiques, au degré d'handicap, à la fatigue, à l'apathie et à la mauvaise perception de son état de santé.

## Introduction

La dépression est une complication fréquente de l´accident vasculaire cérébral (AVC). Sa fréquence est variable et se situe globalement entre 20 et 65% selon les études [1–8]. La dépression post-accident vasculaire cérébral (DPAVC) a une influence négative sur l’évolution des patients en termes de mortalité, de morbidité et de qualité de vie [9]. En Afrique, la fréquence des maladies chroniques non transmissibles telles que l'hypertension artérielle (HTA), les cardiopathies ischémiques (CI) ou les AVC a tendance à augmenter selon les relevés épidémiologiques confirmant ainsi une véritable transition épidémiologique [10]. Cependant, les données concernant la DPAVC demeurent fragmentaires. Elles ne sont pas disponibles pour la République démocratique du Congo (RDC), même si un effort a permis d'identifier les facteurs de risque des AVC tels que l'HTA, le bas niveau socio-économique, les conditions météorologiques et les saisons [11]. Les troubles neuropsychiatriques des AVC dont en particulier la DPAVC ne sont ni diagnostiqués ni pris en charge par les praticiens congolais peu ou pas formés à les reconnaître. La présente étude a pour but de combler cette double lacune épidémiologique et clinique. L'objectif général poursuivi est de décrire le tableau clinique de la dépression post-accident vasculaire cérébral à Kinshasa. A cet effet, deux objectifs spécifiques ont été fixés : évaluer la prévalence de la DPAVC et déterminer les facteurs sociodémographiques et cliniques associés à la DPAVC auprès d'un échantillon des patients d'un centre de réhabilitation à Kinshasa.

## Méthodes

**Figure 1:**
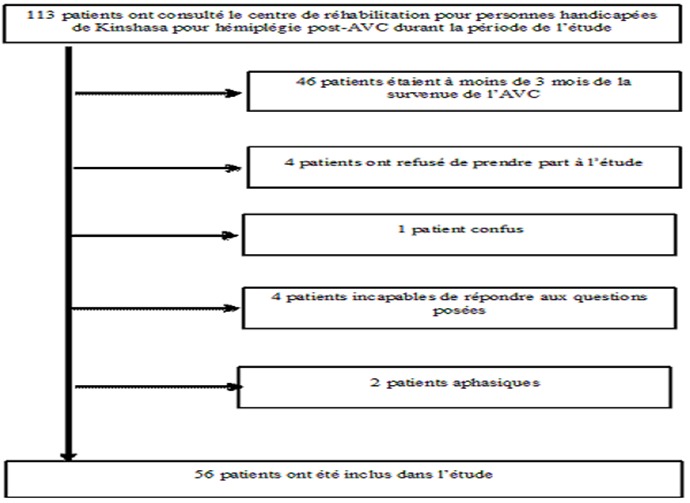
Diagramme de la sélection des patients

**Design et sujets de l’étude**: Il s'agit d'une étude transversale portant sur 56 patients suivis pour hémiplégie post-accident vasculaire cérébral au Centre de réhabilitation pour personnes handicapées de Kinshasa (CRPHK) du 1er au 31 août 2011. Les critères d'inclusion étaient les suivants : donner son consentement éclairé, être âgé de 18 ans ou plus et avoir totalisé au minimum trois mois depuis la survenue de l'AVC au moment de l’étude. Les patients confus ou présentant un trouble profond de la conscience, les patients incapables de comprendre et d'exécuter les ordres ainsi que les patients aphasiques ont été exclus de l’étude (Figure 1).

**Clearance éthique:** Cette étude a été approuvée par la Commission d’éthique biomédicale hospitalo-facultaire de l'Université catholique de Louvain.

**Evaluation clinique des sujets de l’étude:** Chaque participant a bénéficié d'une anamnèse et d'un examen clinique neurologique et psychiatrique. Le diagnostic de la dépression a été posé avec le Patient Health Questionnaire en abrégé PHQ9 [12]. Les informations suivantes ont été recueillies: âge, sexe, situation socioprofessionnelle, niveau d’études, habitude de consommation (alcool, tabac), antécédents psychiatriques, antécédents médicochirurgicaux, durée de la maladie, qualité du sommeil, état clinique neurologique, fatigue [13], invalidité [14], qualité de vie , apathie [15] et résultat du test du Go/No-go [16]. Toutes ces variables étaient considérées comme facteurs prédictifs potentiels du développement d'une DPAVC.

**Analyses statistiques:** Les résultats obtenus ont été dichotomisés (Tableau 1) et analysées avec les logiciels Epi info 6. 04 version française et Stata/IC 11. 2. Dans un premier temps, les résultats des analyses descriptives ont été présentés sous forme de fréquences pour les variables qualitatives. Les variables quantitatives ont été présentées sous forme de médiane et intervalles interquartiles (P25 et P75) pour les variables non normalement distribuées. La moyenne ± écart-type a été utilisée à des fins de comparaison dans la discussion des résultats. Les recherches d´association entre différentes variables ont été réalisées en utilisant les tables de comparaisons des proportions et le test de Chi carré de Pearson, ou le test de Fischer quand les conditions de validité n’étaient pas respectées. Le seuil de signification statistique retenu était de 5%.

**Tableau 1:** Catégories dichotomiques utilisées pour l'analyse des résultats

Variables	Catégories pour l'analyse	Critères
**Dépression**	Minimale à légère	1 ≤ Score PHQ9 ≤ 9
	Modérée à sévère	10 ≤ Score PHQ9 ≤ 27
**Etat clinique neurologique**	Atteinte légère	1 ≤ ScoreNIHSS < 5
	Atteinte modérée à sévère	Score NIHSS ≥ 5
**Fatigue**	Compatible avec la dépression	Score FSS ≥4.5
	Fatigue sans dépression	Score FSS <4.5
**Invalidité**	Capable de marcher sans aide	Score Rankin ≤3
	Incapable de marcher sans aide	Score Rankin >3
**Apathie**	Modérée à sévère	Score Lars ≤ - 17
	Absente ou tendance	Score Lars > -17
**Go/no go**	Pathologique	Temps de réaction élevée
	Non pathologique	Dans les normes

## Résultats

Dans notre échantillon, les hommes étaient plus nombreux parmi les patients enrôlés avec un total de 35 hommes soit 62. 50% (Tableau 2). L’âge moyen était de 54. 45 ±12. 57 ans, avec un minimum de 23 ans et un maximum de 75 ans. L’âge médian était de 56 ans (P25:46 ans ; P75 : 64. 50 ans). Trois patients sur quatre (75. 00%) étaient âgés de moins de 65 ans; 78. 60% des patients consommaient de l'alcool contre 25. 00% pour le tabac. Aucun des sujets de l’étude ne présentait d'antécédents psychiatriques. L'antécédent d'HTA était présent chez 76. 78%, le diabète et les traumatismes crâniens étaient retrouvés chez 17. 86% des patients. L’épilepsie et les cardiopathies étaient moins fréquentes, ont été rapportés respectivement par 2 (3. 57%) et 4 (7. 14%) patients. Les antécédents médicochirurgicaux familiaux comptaient 62,50% d'hypertension artérielle, 5. 40% d'antécédent de cardiopathie, 28. 60 % d'antécédents d'AVC, 7. 14% d'antécédents d’épilepsie et 21. 43% d'antécédents de diabète. Au moment de l’étude, douze patients (21. 43%) présentaient une dépression modérée à sévère. L'AVC était survenu depuis 3 à 6 mois pour 12 patients (21. 43%), 6 à 12 mois pour 10 patients (17. 86%) et plus de 12 mois pour 35 d'entre eux (60. 70%). Le score médian observé à l’échelle NIHSS était de 6,50 (P25 : 4. 00 ; P75 :12. 00). Le score moyen était de 7,98±5,18. Dix-huit patients (32. 14 %) présentaient une atteinte légère contre 38 (67. 86%) avec une atteinte modérée à sévère. La dépression a été évaluée avec le PHQ9. Le score médian observé au PHQ9 était de 5,00 (P25 : 1. 50 ; P75 :8. 50) ; le score moyen était de 5. 79±5. 55. A l’échelle de Rankin, 64. 29% des patients étaient capables de marcher sans aide alors que 35. 71% présentaient un handicap fonctionnel modéré à sévère. A l'autoévaluation visuelle analogique de leur état de santé, 69. 64% des patients donnaient une cote supérieure à cinq sur une échelle de 1 à 10 à leur état de santé. Sur cette échelle, 1 représentant l’état de santé le moins bon possible et 10 le meilleur possible. L'apathie modérée à sévère était présente chez 44. 64%. A l’échelle FSS, le score médian observé était de 1. 0 (P25 :1,00 ; P75 :2. 25). Le score moyen de fatigue observé était de 1. 91±1. 67. Six patients (10. 71%) présentaient un score de fatigue compatible avec la dépression. Vingt-cinq patients (44. 64%) présentaient un résultat pathologique au Go/No-Go. La dépression a été définie comme dépression modérée à sévère selon le PHQ9. Les rapports entre la dépression et les caractéristiques cliniques sont résumés dans le Tableau 3. L'analyse univariée a montré une association statistiquement significative entre un handicap modéré à sévère à l’échelle de Rankin (p=0. 006), l'apathie modérée à sévère (p=0. 003), l'atteinte neurologique modérée à sévère au NIHSS (p=0. 03), un score à l’échelle FSS ≥4. 5 (p<0. 001) (Tableau 3) et l’évaluation mauvaise de l’état de santé sur l’échelle visuelle (p=0. 024).

**Tableau 2:** Caractéristiques sociodémographiques des patients

Paramètres sociodémographiques	n	%
**Sexe**		
Hommes	35	62.50%
Femmes	21	37.50%
**Age en années**		
15-24	1	1.80%
25-34	5	8.90%
35-44	4	7.10%
45-54	17	30.40%
55-64	15	26.80%
65-74	13	23.20%
75-84	1	1.80%
**Niveau d’étude**		
Sans diplôme	8	14.30%
Primaire	17	30.40%
Secondaire	15	26.80%
Graduat(1)	6	10.70%
Licence ou plus(2)	10	17.90%
**Religion**		
Catholique	27	48.20%
Protestant	7	12.50%
Kimbanguiste	2	3.60%
Musulman	1	1.80%
Réveil	13	23.20%
Autres	6	10.70%
**Alcool**		
Présence	44	78.60%
Absence	12	21.40%
**Tabac**		
Présence	14	25.00%
Absence	42	75.00%

1 Graduat: premier cycle d'enseignement supérieur ou universitaire en RD Congo

2 Licence : deuxième cycle d'enseignement supérieur ou universitaire en RD Congo

**Tableau 3:** Rapports entre la dépression et les caractéristiques cliniques

Paramètres Cliniques	Modalités	Dépression		OR (IC 95%)	P
		n	%		
**Antécédent d'HTA**	Absent	4	30.8	1	0.281
	Présent	8	18.60	0.51 (0.12-2.09)	
**Antécédent de diabète**	Absent	11	23.91	1	0.308
	Présent	1	10	0.35 (0.04-3.11)	
**Antécédent d'HTA familiaux**	Absent	4	19	1	0.506
	Présent	8	22.86	1.26 (0.32-4.83)	
**Antécédents familiaux de diabète**	Absent	10	22.72	1	0.496
	Présent	2	16.67	0.68 (0.12-3.63)	
**Echelle NIHSS**	Légère	0	0	1	0.005
	Modérée + sévère	12	31.57	indéfini	
**Rankin**	Capable de marcher sans aide	4	11.11	1	0.016
	Incapable de marcher sans aide	8	40	5.33 (1.35-21.02)	
**Durée maladie**	Durée <6 mois	3	25	1	0.504
	Durée ≥ 6 mois	9	20.45	0.77 (0.18-3.45)	
**Score FSS**	Fatigue sans dépression (<4.5)	6	14	1	<0.001
	Compatible avec la dépression (≥4.5)	5	83.33	30.71 (3.10-303.56)	
**Apathie**	Absente ou légère	2	6.45	1	0,003
	Modérée + sévère	9	37.5	8.70 (1.66-45.48)	
**État de santé autoévalué**	Bon	5	12.82	1	0.024
	Mauvais	7	41.17	4.76 (1.24-18.31)	
**Go / No Go**	Normale	7	24.00	1	0.737
	Pathologique	5	17.86	1.53 (0.42-5.57)	

## Discussion

Le présent travail est le premier à décrire profil clinique de la DPAVC à Kinshasa (RD Congo). Il est l'un des rares du genre mené en Afrique subsaharienne. Il a la particularité d'avoir les patients en phase de réhabilitation, à distance de la survenue de la maladie, au moment où les patients reprennent leur vie dans la communauté. La fréquence observée est de 21. 43%. La prévalence de la DPAVC varie selon les études en raison de la variabilité des caractéristiques des sujets inclus et des critères-diagnostics utilisés. Le résultat observé dans la présente étude se rapproche de ceux trouvés dans les travaux publiés dans le monde. El Husseini et al, qui ont évalué les patients à 3 mois et à 12 mois en post-AVC avec le PHQ8 ont rapporté une fréquence de 17. 9% et de 16. 4% [4]. L’étude de White et al. portant sur 2477 patients présentant des lacunes 4 mois après la survenue de l'AVC et évalués avec le PHQ 9 a rapporté un taux de 19% [5]. Oladiji et al. ont rapporté une fréquence de 25% des patients déprimés sur un total de 51 à Lagos [6]. En 2007, Barker-Collo a rapporté une prévalence de 22. 8% 3 mois après la survenue de l'AVC auprès d'un échantillon de 73 patients [7]. En 2002, dans une étude portant sur des patients 3 à 6 mois après la survenue de l'AVC, Glamcevski II et al. ont rapporté un taux de 15% des cas de dépression modéré à sévère chez des patients malaisiens [8]. Dans l’étude Interstroke, l'HTA était considérée comme le facteur de risque modifiable le plus important dans la survenue des AVC [17]. L'antécédent de HTA a été retrouvé chez 76. 78% des patients. En même temps 62. 50% des patients ont rapporté un antécédent familial d'HTA. Contrairement à des auteurs comme Tennen et al. ou White et al, nous n'avons pas observé de relation statistiquement significative entre la survenue de la DPAVC et l'antécédent d'HTA [5, 18]. Ceci peut s'expliquer par la faible taille de notre échantillon (56 patients). L’étude de White et al. comptait 2477 participants et celle de Tennen et al. 107 sujets. Le score moyen obtenu au NIHSS était de 8. 26±5. 50 est comparable aux valeurs observées ailleurs chez des patients évalués à distance de la survenue de l'AVC [19]. Le nombre des patients capables de marcher sans aide (MRS 0-3) était de 37 soit 66. 07% du total. Dans une étude menée au Malawi portant sur un total de 147 patients, le pourcentage des patients capables de marcher sans aide (MRS0-3) était de 46. 3% à 6 mois et de 41. 5% à une année. La différence observée pourrait s'expliquer par la taille de l’échantillon, les caractéristiques de la population étant plutôt comparables à l'exception du fait que 34% des patients malawites étaient séropositifs au VIH. Cependant, l’étude malawite avait montré que la séropositivité des patients n'avait pas d'influence sur les conséquences cliniques de l'AVC [20]. L'apathie modérée à sévère a été retrouvée chez 44. 64% des patients. La fréquence observée dépasse largement celle rapportée dans la littérature. Jorge et al. dans une récente revue de littérature rapportent une fréquence variant entre 20 et 25% [21]. Cet écart pourrait être lié aux différences dans les méthodes et le recrutement des patients. Dans la présente étude, l'apathie a été évaluée avec le Lille Apathy rating Scale. Des travaux ultérieurs sur les populations africaines semblent indiqués pour confirmer cet écart par rapport aux données actuelles de la littérature. La dépression était plus fréquente chez les patients présentant une atteinte neurologique modérée à sévère au NIHSS (p=0. 03), incapables de marcher sans aide au MRS (p=0. 01), avec un score ‘4. 5 à l’échelle de fatigue (p=0. 0008), présentant une apathie modérée à sévère au LARS (0. 006) et chez les patients ayant spontanément évalué leur état de santé comme étant mauvais sur une échelle visuelle (p=0. 024). Cependant, Marasco et al. N'ont pas observé de relation statistiquement significative entre la dépression et la sévérité de l'atteinte neurologique chez 54 patients hospitalisés dans une unité des soins sémi-intensifs [22]. Il s'agissait dans leur étude d'un échantillon évalué en phase aiguë, à la différence de notre échantillon dont les sujets ont été évalués à distance de la survenue de l'AVC c´est-à-dire en phase chronique. Il semble en effet que la relation entre le degré d'atteinte neurologique et la survenue de la DPAVC comme nous l'avons observé soit plus claire en phase chronique même si l'on peut déjà la retrouver en phase aiguë [23–30]. Plusieurs travaux ont montré le lien entre l'invalidité ou le handicap fonctionnel et la survenue de la DPAVC [31–35]. Ce lien a également été observé dans le présent travail où la dépression était plus fréquente chez les patients incapables de marcher seuls.

La fatigue est fréquemment observée en post-AVC [36]. Glader et al. ont évalué sa fréquence à environ 30% jusqu’à deux ans après la survenue de l'AVC [37]. Le lien observé dans le présent travail entre la fatigue post-AVC et la DPAVC correspond aux données de la littérature. La fatigue en post-AVC est très souvent associée à la dépression [36–39]. Il n'est cependant pas impossible d'observer des cas des patients présentant de la fatigue sans la dépression [40, 41]. Néanmoins, la force de l'association entre la dépression et la fatigue observée dans la présente étude suggère que cette dernière est un indicateur utile pour le clinicien dans le diagnostic de la DPAVC.

L'apathie modérée à sévère était également associée de manière significative à la DPAVC corroborant ainsi les données de littérature [21, 42]. L'association entre l'apathie et la dépression post-AVC est l'objet d'un intérêt croissant de la part des chercheurs. Dans une récente publication, Hama et al. ont proposé de considérer l'entité nosologique DPAVC comme étant un ensemble de deux groupes de symptômes ou syndromes, la DPAVC affective et la DPVC apathique avec peut-être des soubassements neuroanatomiques spécifiques pour chacun qui resteraient à découvrir [43].

La dépression n’était pas associée aux antécédents, à la durée de la maladie ni aux performances réalisées au test Go/No-Go. La littérature rapporte des contre-performances chez les patients porteurs des lésions frontales [44] et chez les patients déprimés [45]. Dans le cadre de cette étude, le test d’évaluation de l´attention n´a pas été discriminant. Des études ultérieures sont souhaitables pour confirmer cette observation.

## Conclusion

La DPAVC est fréquente à Kinshasa. La fréquence observée est comparable à celles observées dans des travaux antérieurs dans le monde et en Afrique. Elle est associée de manière significative à la sévérité des troubles neurologiques évaluées au NIHSS, au degré d´handicap évalué à l´échelle de Rankin, à la fatigue évaluée à l´échelle de sévérité de la fatigue, à l´apathie évaluée l´échelle d´apathie de Lille à la mauvaise perception de son état de santé. Par contre, elle n´est pas associée aux antécédents médicochirurgicaux, à la durée de la maladie ni aux résultats du test Go/No Go, Ces résultats contribuent à une meilleure connaissance de la clinique de la DPAVC dans les populations africaines. Elles permettraient aux médecins traitants de discriminer les patients exposés à la DPAVC.

## References

[1] De Ryck A, Brouns R, Fransen E, Geurden M (2013). A prospective study on the prevalence and risk factors of poststroke depression. Cerebrovasc Dis Extra..

[2] Napon C, Kaboré A, Kaboré J (2012). La dépression post-accident vasculaire cérébral au Burkina Faso. Pan Afr Med J..

[3] Altieri M, Maestrini I, Mercurio A, Troisi P (2012). Depression after minor stroke: prevalence and predictors. Eur J Neurol..

[4] El Husseini N, Goldstein LB, Peterson ED, Zhao X (2012). Depression and antidepressant use after Stroke and transient ischemic attack. Stroke..

[5] White CL, McClure LA, Wallace PM, Braimah J (2011). The correlate and course of depression in patients with lacunar stroke: results from the secondary Prevention of subcortical strokes (SPS3 study). Cerebrovasc Dis..

[6] Oladiji JO, Sra A, Aina FA, Aiyejusunle CB (2009). Risk factors of post-stroke depression among stroke survivors in Lagos, Nigeria. Afr J Psychiatry (Johannesbg)..

[7] Barker-Collo SL (2007). Depression and anxiety 3 months post stroke: Prevalence and correlates. Arch Clin Neuropsychol..

[8] Glamcevski MT, Lynne C, Mcarthur LC, Chong HT (2002). Factors associated with post-stroke depression, a Malaysian study. Neurol J Southeast Asia..

[9] Salter KL, Foley NC, Zhu L, Jutai JW (2013). Prevention of Poststroke Depression: Does Prophylactic Pharmacotherapy Work. J Stroke Cerebrovasc Dis..

[10] Dalal S, Beunza JJ, Volmink J, Adebamowo C (2011). Non-communicable diseases in sub-Saharan Africa: what we know now. Int J Epidemiol..

[11] Longo-Mbenza B, Phanzu-Mbete LB, M'Buyamba-Kabangu JR, Tonduangu K (1999). Hematocrit and stroke in black Africans under tropical climate and meteorological influence. Ann Med Interne (Paris)..

[12] Carballeira Y, Dumont P, Borgacci S, Rentsch D (2007). Criterion validity of the French version of Patient Health Questionnaire (PHQ) in a hospital department of internal medicine. Psychol Psychother..

[13] Valko PO, Bassetti CL, Bloch KE, Held U (2008). Validation of the fatigue severity scale in a Swiss cohort. Sleep..

[14] Huybrechts KF, Caro JJ, Xenakis JJ, Vemmos KN (2008). The prognostic value of the modified Rankin Scale score for long-term survival after first-ever stroke. Results from the Athens Stroke Registry. Cerebrovasc Dis..

[15] Dujardin K (2010). Échelle lilloise d'apathie - Lille apathy rating scale (LARS). Pratique Neurologique- FMC..

[16] Sternberg S, Koster WG (1969). The discovery of processing stages: Extensions of Donders` Method. Attention and performance II.

[17] O'Donnell MJ, Xavier D, Liu L, Zhang H (2010). Risk factors for ischaemic and intracerebral haemorrhagic stroke in 22 countries (the INTERSTROKE study): a case-control study. Lancet..

[18] Tennen G, Hermann N, Black SE, Levys KS (2011). Are vascular risk factors associated with Post-stroke depression. J Geriatr Psychiatry Neurol..

[19] Meyer BC, Raman R, Ernstrom K, Tafreshi GM (2012). Assessment of Long Term Outcomes for the STRokE DOC Telemedicine Trial (STRokE DOC-LTO). J Stroke Cerebrovasc Dis..

[20] Heikinheimo T, Chimbayo D, Kumwenda JJ, Kampondeni S (2012). Stroke Outcomes in Malawi, a Country with High Prevalence of HIV: A Prospective Follow-Up Study. PLoS One..

[21] Jorge RE, Starkstein SE, Robinson RG (2010). Apathy following stroke. Can J Psychiatry..

[22] Marasco G, Iavarone A, Ronga B, Martini V (2011). Depressive symptoms in patients admitted to a semi-intensive Stroke Unit. Acta Neurol Belg..

[23] Berg A, Palomaki H, Lehtihalmes M, Phil L (2003). Poststroke depression: an 18-month follow-up. Stroke..

[24] Pohjasvaara T, Leppavuori A, Siira I, Vataja R (1998). Frequency and clinical determinants of poststroke depression. Stroke..

[25] Hermann N, Black Se, Lawrence J, Szekely C (1998). The Sunnybrook Stroke Study: a prospective study of depressive symptoms and functional outcome. Stroke..

[26] Singh A, Black SE, Hermann N, Leibovitch FS (2000). Functional and neuroanatomic correlations in poststroke depression. Stroke..

[27] Sienkiewicz-Jarosz H, Milewska D, Bochy'ska A, Che'mniak A (2010). Predictors of depressive symptoms in patients with stroke- a three-month follow-up. Neurol Neurochir Pol..

[28] Hackett ML, Anderson CS (2005). Predictors of Depression after Stroke: A Systematic Review of Observational Studies. Stroke..

[29] Burvill P, Johnson G, Jamrozik K, Anderson C (1997). Risk Factors For Post-Stroke Depression. Int J Geriatr Psychiatry..

[30] Choi-Kwon S, Han K, Choi S, Suh M (2012). Poststroke depression and emotional incontinence: Factors related to acute and subacute stages. Neurology..

[31] Chau JP, Thompson DR, Chang AM, Woo J (2010). Depression among Chinese stroke survivors six months after discharge from a rehabilitation hospital. J Clin Nurs..

[32] Robinson RG, Spalletta G (2010). Poststroke depression: a review. Can J Psychiatry..

[33] Carod-Artal FJ, Ferreira Coral L, Trizotto DS, Menezes Moreira C (2009). Poststroke depression: prevalence and determinants in Brazilian stroke patients. Cerebrovasc Dis..

[34] Pandian JD, Kaur A, Jyotsna R, Sylaja PN (2012). Complications in Acute Stroke in India (CAST-I): A Multicenter Study. J Stroke Cerebrovasc Dis..

[35] Nys GM, van Zandvoort MJ, van der Worp HB, de Haan EH (2005). Early depressive symptoms after stroke: neuropsychological correlates and lesion characteristics. J Neurol Sci..

[36] De Groot MH, Phillips SJ, Eskes GA (2003). Fatigue Associated With Stroke and Other Neurologic Conditions: Implications for Stroke Rehabilitation. Arch Phys Med Rehabil..

[37] Glader EL, Stegmayr B, Asplund K (2002). Poststroke Fatigue: A 2-Year Follow-Up Study of Stroke Patients in Sweden. Stroke..

[38] Schepers V, Visser-Meily A, Ketelaar M, Lindeman E (2006). Post-stroke fatigue: course and its relation to personal and stroke-related factors. Arch Phys Med Rehabil..

[39] Van de Port IGL, Kwakkel G, Bruin M, Lindeman E (2007). Determinants of depression in chronic stroke: a prospective cohort study. Disability and Rehabilitation..

[40] Staub F, Bogousslavsky J (2001). Post-Stroke Depression or Fatigue. Eur Neurol..

[41] Colle F, Bonan I, Gellez Leman MC (2006). Fatigue après accident vasculaire cérébral. Ann Readapt Med Phys.

[42] Withall A, Brodaty H, Altendorf A, Sachdev PS (2011). A longitudinal study examining the independence of apathy and depression after stroke: the Sydney Stroke Study. Int Psychogeriatr..

[43] Hama S, Yamashita H, Yamawaki S, Kurisu K (2011). Post-stroke depression and apathy: Interactions between functional recovery, lesion location, and emotional response. Psychogeriatrics..

[44] Mouchabac S (2009). Comportements impulsifs, agressivité et oxyde nitrique. Tendances et Débats..

[45] Erickson K, Drevets WC, Clark L, Cannon DM (2005). Mood-Congruent Bias in Affective Go/No-Go Performance of Unmedicated Patients With Major Depressive Disorder. Am J Psychiatry..

